# Robert H. Rubin and infectious disease in transplantation

**DOI:** 10.3389/frtra.2023.1315397

**Published:** 2023-11-21

**Authors:** Jay A. Fishman

**Affiliations:** Transplant Infectious Disease and Compromised Host Program and Transplant Center, Massachusetts General Hospital, Harvard Medical School, Boston, MA, United States

**Keywords:** Robert Rubin, infectious diseases, transplantation, physician-scientist, immune suppression

## Abstract

Robert H. Rubin, M.D. was among the first physician-scientists to focus attention on the infectious diseases associated with immune suppression. A superb bedside clinician and teacher, he developed many of the concepts central to the care and improved survival of organ transplant and stem cell transplant recipients. These concepts have provided the basis of clinical investigation and basic research for multiple generations of infectious disease specialists, immunologists, and transplant surgeons.

No one who practices organ transplantation is untouched by the contributions of Robert H. Rubin as a visionary in the practice and teaching of science and medicine. Dr. Rubin was a graduate of Williams College and Harvard Medical School. After medical residency at the Peter Bent Brigham Hospital, he was a medical intelligence officer at the Center for Disease Control. He completed his infectious disease training at the Massachusetts General Hospital (MGH) and Tufts Medical Center. Dr. Rubin was Chief of Transplant Infectious Disease at the MGH for over twenty years before becoming Clinical Director of Infectious Disease at the Brigham and Women's Hospital (Figure 1). Early in his time at MGH, he co-directed the Medical Intensive Care Unit with his wife, Dr. Nina Tolkoff-Rubin (who he described as the “best doctor” he ever met). He also was among the first faculty in the joint Harvard Medical School (HMS)-Massachusetts Institute of Technology (MIT) Division of Health Sciences and Technology (HST) program, and he would ultimately become the Gordon and Marjorie Osborne Professor of Health Sciences and Technology in the Harvard-Massachusetts Institute of Technology Division of Health Sciences and Technology. In 1995 he was the first internist appointed as Microbiologist in the Department of Surgery at MGH in recognition of his role in the developing organ transplant program. He became a Professor of Medicine at Harvard Medical School in 1999.

**Figure 1:**
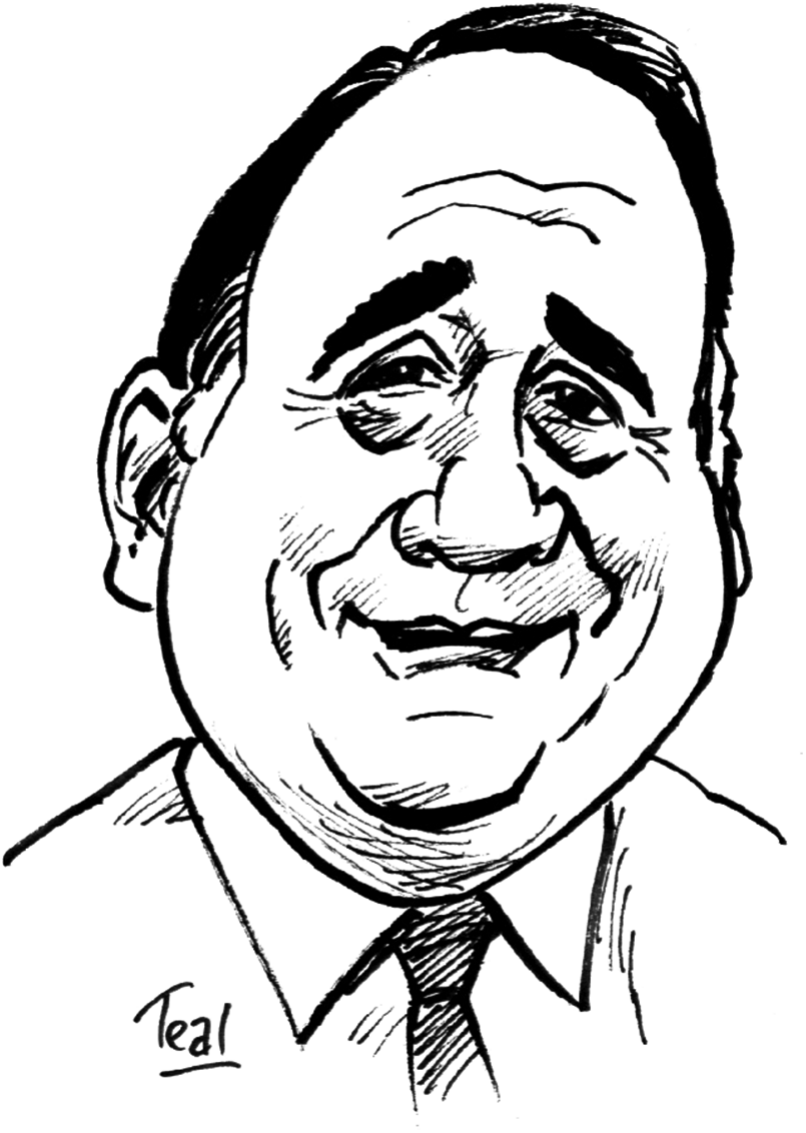
Robert H. Rubin as drawn for the President's Address, Jay A. Fishman, M.D., American Society of Transplantation, Seattle, WA, 2005.

Dr. Rubin's studies at the interface of technology, bedside clinical care and research drug development and business provided a platform for development of innovative and interdisciplinary programs in science and technology, in clinical investigation at Harvard and MIT, but also at the Sloan School of Business and at the Harvard School of Public Health. He developed (with Professor Christopher Walsh) an elective course in clinical pharmacology and therapeutics for which he received multiple teaching awards. The Clinical Investigator Training Program, with Dr. Alan Moses, was a program in clinical research which trained dozens of young physician-scientists and provided a Master of Medical Science degree in Clinical Investigation. The didactic materials for this program became the basis for training programs in clinical investigation in Israel and Latin America. In each venue, he developed a new group of young minds who could work across traditional boundaries—much as he did himself- and speed the advancement of medical knowledge to the bedside. His pioneering studies of radiological imaging of infectious disease and inflammatory processes (the Rubin scans”) quickly became part of clinical care. Bob wrote over 400 original reports, reviews, chapters, and textbooks. Some of his early papers are classics of diagnosis and new approaches to thinking about mechanisms of disease and therapy.

The skills and concepts developed at the bedside lead him to initiate and chair committees on Transplant Infectious Disease for the Transplantation Society, the Immunocompromised Host Society, the American Society of Transplant Physicians and in a series of International Conferences on Transplantation Infectious Disease. His approaches were captured in a book, co-edited with Dr. Lowell Young, Clinical Approach to Infection in the Compromised Host and as Editor-in-Chief of *Transplant Infectious Disease*, an official journal of the Transplantation Society.

Bob was best known for his skills as a teacher and clinician. Twice each day, Transplant Infectious Disease physicians would round with the abdominal transplant teams—adjusting immunosuppression, organizing diagnostic procedures, selecting antibiotics, and arguing about patient management with expert transplant surgeons, nephrologists, hepatologists as well as bench immunologists and pathologists. Discussions were often “intense”. At the bedside, he was an instinctual clinician for the sickest and most complex patients. He focused attention on the “net state of immunodeficiency”, a conceptual framework for all the host factors that contribute to infectious risk—as well as the epidemiological exposures that defined the specific organisms causing infection in each community—known as “the sentinel chicken” (as opposed to the canary of coal mines). His “equation” of the “semi-quantitative relationship” between immunosuppression and infectious exposures is taken as gospel—any intervention that reduces risk (vaccines, prophylaxis) allows greater degrees of immunosuppression and lowers the risk for graft rejection. Conversely, infection became a measure of the technical skill of the surgical team and the intensity of immunosuppression.

His rigor as a bedside clinician and teacher led to the development of most of the diagnostic paradigms for infection and the evolution of most current prophylactic and therapeutic regimens for infection in transplantation. Bob's creative juices were stimulated by unexplained fevers and syndromes in these immunosuppressed patients. At first, his contributions were diagnostic and epidemiologic—discovering outbreaks of Legionnaires disease and *Pseudomonas* transmitted by ventilation ducts wafting bacteria over transplant recipients. Then, he “discovered” cytomegalovirus—CMV—and began to characterize the syndrome that has tormented transplanters since the beginning of the field (1–4). His efforts led, in part, to the clinical development of fluconazole and ganciclovir. These observations resulted in his characterization of the “net state of immunodeficiency” and the “timeline of post-transplant infections” that have guided the development of prophylactic regimens (e.g., for urinary tract infection, Pneumocystis and CMV) and diagnosis (infections at the “wrong time” indicating unusual epidemiologic hazard or excessive immunosuppression) (5–8). These are now considered among the gospels of transplantation. He also used a broad understanding of science and medicine to make predictions about the mechanisms of multiple important clinical syndromes—CMV and other viral infections—and the modulation of immunosuppression as a part of the “therapeutic prescription” required for the successful eradication of infections in these susceptible hosts. His intuitive understanding of the multiple effects of viruses—opportunistic infections, graft rejection, malignancy—are only now being elucidated at the molecular level. His legacy is immense.

He was an amazing teacher to 1,000's of students. He was a sponge for new knowledge. He set impossibly high standards. All with the goal of training a generation of inquisitive clinician-scientists and accelerating the speed and relevance of the advancement of medical knowledge. He had the ability to synthesize and explain complex concepts. What did he teach? This was where some of Bob's unique skills emerged. Bob was never constrained by the usual intellectual barriers. He was never impressed by the memorization of lists of diseases and therapies. He always wanted to know how to do everything better—for family and friends and for our patients. Bob had a clear vision of the level of performance needed to optimize the clinical care of complex patients. We participated in some of the earliest organ transplants in HIV-infected individuals—in the pre-HARRT era—with predictably poor outcomes. He routinely supported talented female and minority trainees before this was even entertained elsewhere. We went to the OR to observe and ended up holding retractors and suction—at a time when transplant programs were understaffed. He obtained new antimicrobials for patients with viral and fungal infections—and developed regimens for prophylaxis and therapy that remain standards today. Bob was fond of quoting Dr. Louis Weinstein—one of his teachers—as saying “There are only three things we don't know about antimicrobial therapy: which drug to use, how much and for how long”.

Bob began to pose questions based on his experience as a clinician and epidemiologist. How do we use organs from infected donors? Which other latent infections became activated in the transplant recipient? Why was graft and patient survival less good in patients infected with CMV (“indirect effects”)? And what was the role of other viruses in transplant outcomes? The questions he posed have guided the evolution of research into host-pathogen relationships, clinical pharmacology, and host defense mechanisms. His teaching has, without question, saved many lives among our most susceptible patients. Robert Rubin cared deeply about several things—family and friends, his patients, and new ideas. His extraordinary efforts were among the first aimed at the advancement of the translational science and clinical knowledge within our field. We are all better for having experienced his passion and skill. By force of personality, Bob was destined to be a leader.

Perhaps a vignette captures some of the essence of Robert Rubin as a mentor.

It's Tuesday. Life seems under control as an ID Fellow. My beeper goes off. Bob is away. He calls from the plane: “You’re covering. Everyone is fine. Oh, and I need you to give a talk for me”. As in most clinical spheres, things didn't always go smoothly—but he defended my efforts strenuously. He left me a box of slides on his way out the door—in a few days, I am supposed to give a talk for Bob Rubin—on another continent. I'd been in this situation many times before. The talk was always on a topic on which Bob was a world's expert, and for which I would only generously be considered an amateur—to an audience which is expecting Bob. So, off I would go to great and far-away places like Venice, Rio, London, Sydney, Vancouver. There was no point of discussion. Bob deferred to no one except Nina and their daughter, Melissa. Once you joined the Rubin clan you were “all in”. Thus, Bob (and Nina and Melissa) decided that my family (not me) needed a Golden retriever—I refused as I was “too busy”—so he bought us our first dog anyway—not for me, but for my family—as I “had no judgement worth noting in this area”. As usual, he was right. Bob was always right.

## Data Availability

The original contributions presented in the study are included in the article/Supplementary Material, further inquiries can be directed to the corresponding author.
